# Impact of Chronic Kidney Disease Severity on Clinical Outcomes After Drug‐Eluting Stent Implantation: A Propensity Score–Matched Analysis

**DOI:** 10.1002/clc.70387

**Published:** 2026-06-19

**Authors:** Hongxu Zhu, Qi Jin

**Affiliations:** ^1^ Department of Cardiology Beijing Haidian Hospital Beijing China

**Keywords:** chronic kidney disease, drug‐eluting stent, major adverse cardiovascular events, percutaneous coronary intervention, propensity score matching

## Abstract

**Background:**

Chronic kidney disease (CKD) is prevalent among patients undergoing percutaneous coronary intervention (PCI) and associated with adverse cardiovascular outcomes. This study evaluated the association between moderate‐to‐severe CKD and major adverse cardiovascular events (MACE) after drug‐eluting stent (DES) implantation using propensity score matching.

**Methods:**

This retrospective cohort study included 200 patients undergoing PCI with DES implantation (January 2020 to January 2025). Patients were stratified by baseline eGFR: non‐CKD/mild CKD (≥ 60 mL/min/1.73 m^2^); and moderate‐to‐severe CKD (< 60 mL/min/1.73 m^2^). After 1:1 propensity score matching, 55 pairs were analyzed. The primary endpoint was MACE, defined as all‐cause death, myocardial infarction, stroke, or ischemia‐driven repeat revascularization.

**Results:**

After matching, patients with moderate‐to‐severe CKD had lower MACE‐free survival (log‐rank *p* = 0.003). Cox regression with robust sandwich standard errors clustered by matched pair showed that moderate‐to‐severe CKD was independently associated with higher MACE risk (HR 3.20, 95% CI 1.36–7.53; *p* = 0.008). Renal function deterioration (eGFR decline ≥ 30%) occurred more frequently in the CKD group (20.0% vs. 3.6%; *p* = 0.028). Higher rates of BARC type 2–5 bleeding and contrast‐associated AKI were observed in the CKD group; contrast‐associated AKI reached statistical significance in the matched analysis (*p* = 0.030). Sensitivity analysis using inverse probability of treatment weighting supported the robustness of these findings (MACE: *p* < 0.001).

**Conclusions:**

Moderate‐to‐severe CKD was independently associated with a higher risk of MACE and renal deterioration after DES implantation. These findings support incorporating CKD severity into post‐PCI risk stratification and highlight the potential value of integrated cardiorenal monitoring and individualized management in this high‐risk population.

## Graphical abstract

Moderate‐to‐severe CKD was associated with higher MACE and renal function deterioration after DES implantation in a propensity score‐matched cohort, supporting CKD‐based cardiorenal risk stratification and closer post‐PCI monitoring.

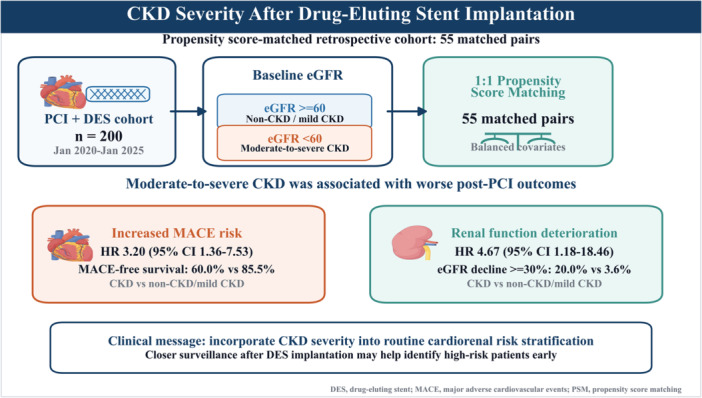

**Abbreviations**
ACEI/ARB: angiotensin‐converting enzyme inhibitor/angiotensin receptor blocker
AKI: acute kidney injury
BARC: Bleeding Academic Research Consortium
BMI: body mass index
CABG: coronary artery bypass grafting
CAD: coronary artery disease
CA‐AKI: contrast‐associated acute kidney injury
CI: confidence interval
CKD: chronic kidney disease
CTO: chronic total occlusion
DAPT: dual antiplatelet therapy
DES: drug‐eluting stent
eGFR: estimated glomerular filtration rate
HR: hazard ratio
IPTW: inverse probability of treatment weighting
KDIGO: Kidney Disease: Improving Global Outcomes
LVEF: left ventricular ejection fraction
MACE: major adverse cardiovascular events
MI: myocardial infarction
OAC: oral anticoagulant
PCI: percutaneous coronary intervention
PS: propensity score
PSM: propensity score matching
SD: standard deviation
SGLT2i: sodium‐glucose cotransporter‐2 inhibitor
SMD: standardized mean difference
TLR: target lesion revascularization.

## 1 Introduction

Coronary artery disease (CAD) remains a leading cause of morbidity and mortality worldwide, accounting for approximately 9 million deaths annually [1]. Percutaneous coronary intervention (PCI) with drug‐eluting stent (DES) implantation has revolutionized the management of obstructive CAD and has become the predominant revascularization strategy for eligible patients [2]. Over the past decades, advances in stent technology, adjunctive pharmacotherapy, and procedural techniques have substantially improved outcomes following PCI. Nevertheless, a considerable proportion of patients undergoing PCI harbor comorbidities that adversely affect prognosis. Among these, chronic kidney disease (CKD) has emerged as a particularly important determinant of clinical outcomes. Data indicate that approximately 30% to 40% of patients presenting for coronary revascularization have concomitant CKD, defined as an estimated glomerular filtration rate (eGFR) below 60 mL/min/1.73 m^2^ [3]. This high prevalence reflects the shared risk factors between CKD and CAD, including diabetes mellitus, hypertension, and advanced age [4]. The coexistence of these conditions creates a high‐risk phenotype with important implications for post‐PCI management and outcomes.

Patients with CKD undergoing PCI face a paradox of heightened risk for both ischemic and hemorrhagic complications [5, 6]. Reduced kidney function has been consistently associated with increased rates of MACE, including all‐cause mortality, myocardial infarction (MI), stent thrombosis, and need for repeat revascularization following DES implantation [7, 8]. Concurrently, CKD patients exhibit altered platelet function and coagulation abnormalities that elevate the risk of bleeding complications during and after PCI [9]. Furthermore, exposure to contrast media during coronary angiography places these patients at considerable risk for contrast‐associated acute kidney injury (CA‐AKI), potentially accelerating the progression of underlying renal disease [10]. This dual vulnerability creates substantial challenges in clinical decision‐making and optimal management strategies.

Despite the high prevalence of CKD among patients undergoing coronary revascularization, evidence guiding treatment strategies in this population remains limited. Most randomized controlled trials evaluating DES efficacy have historically excluded patients with advanced CKD, resulting in a paucity of data directly applicable to this high‐risk subgroup [11]. Existing observational studies have yielded inconsistent conclusions regarding the prognostic impact of varying degrees of renal impairment on post‐PCI outcomes, partly attributable to heterogeneous study designs, variable definitions of CKD severity, and inadequate adjustment for confounding variables [12]. The prognostic association between CKD severity and the balance of cardiovascular efficacy and safety outcomes following DES implantation warrants further investigation.

Accordingly, this retrospective cohort study aimed to characterize the multidimensional cardiovascular, bleeding, and renal risk associated with CKD severity in patients undergoing contemporary DES implantation. Using propensity score matching complemented by inverse probability of treatment weighting (IPTW) sensitivity analysis to minimize selection bias and confounding, we compared MACE incidence and safety outcomes between patients with moderate‐to‐severe CKD (eGFR < 60 mL/min/1.73 m^2^) and those with preserved or mildly reduced kidney function (eGFR ≥ 60 mL/min/1.73 m^2^).

## 2 Material and Methods

### 2.1 Study Design

**Figure 1:**
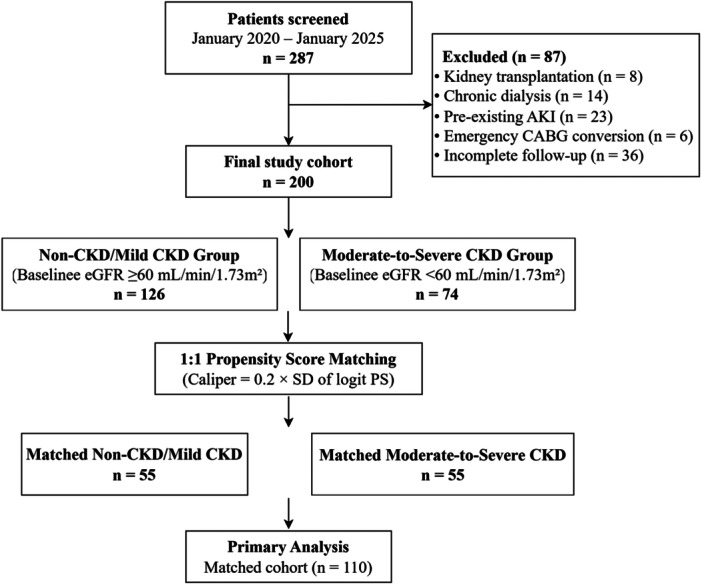
Patient selection flowchart. AKI, acute kidney injury; CABG, coronary artery bypass grafting; CKD, chronic kidney disease; eGFR, estimated glomerular filtration rate.

This single‐center retrospective cohort study was conducted at Haidian Hospital, Beijing, China. Consecutive patients who underwent PCI with DES implantation between January 2020 and January 2025 were screened for eligibility (Figure 1). The study protocol was approved by the Institutional Ethics Committee of Haidian Hospital (Approval No.: M202620), and the requirement for individual informed consent was waived given the retrospective nature of the analysis. This study was conducted in accordance with the ethical principles outlined in the Declaration of Helsinki.

### 2.2 Study Population

Patients were eligible for inclusion if they met all of the following criteria: (1) age ≥ 18 years at the time of the index procedure; (2) underwent PCI with at least one DES implantation during the study period; and (3) had available baseline serum creatinine measurements within 7 days prior to the index procedure. Patients were excluded if they met any of the following criteria: (1) history of kidney transplantation; (2) receiving chronic maintenance dialysis (hemodialysis or peritoneal dialysis for >3 months); (3) conversion to emergency coronary artery bypass grafting (CABG) during the index hospitalization; (4) evidence of pre‐existing AKI at baseline, defined according to the Kidney Disease: Improving Global Outcomes (KDIGO) criteria as an increase in serum creatinine ≥ 0.3 mg/dL within 48 h or ≥1.5 times from baseline within the preceding 7 days [13]; or (5) incomplete follow‐up data (loss to follow‐up before the primary endpoint ascertainment).

### 2.3 Group Definition and eGFR Calculation

The eGFR was calculated using the 2021 CKD Epidemiology Collaboration creatinine equation without the race coefficient, as recommended by the National Kidney Foundation and the American Society of Nephrology Task Force [14, 15]: eGFR = 142 × min(Scr/κ, 1)^α × max(Scr/κ, 1)^ − 1.200 × 0.9938^Age × 1.012 [if female], where Scr is serum creatinine (mg/dL), κ is 0.7 for females and 0.9 for males, *α* is −0.241 for females and −0.302 for males, min indicates the minimum of Scr/κ or 1, and max indicates the maximum of Scr/κ or 1.

Patients were stratified into two groups based on baseline eGFR: (1) non‐CKD/mild CKD group (eGFR ≥ 60 mL/min/1.73 m^2^, corresponding to CKD stages 1–2 or no CKD) and (2) moderate‐to‐severe CKD group (eGFR < 60 mL/min/1.73 m^2^, operationally defined by baseline eGFR and corresponding to CKD stages 3–5 per KDIGO staging), according to the KDIGO 2024 Clinical Practice Guideline for CKD staging [16]. Given the retrospective design, CKD chronicity could not be formally confirmed; group classification was therefore operationally based on a single baseline eGFR measurement.

### 2.4 Data Collection

Baseline demographic characteristics, clinical history, laboratory parameters, angiographic findings, and procedural details were extracted from the electronic medical record system. The following variables were collected: age, sex, body mass index, hypertension, diabetes mellitus, dyslipidemia, prior MI, prior PCI or CABG, heart failure, left ventricular ejection fraction (LVEF), clinical presentation, and hemoglobin. Angiographic variables included the number of diseased vessels, presence of left main CAD, chronic total occlusion (CTO), and multivessel disease. Procedural and angiographic variables included access site (radial or femoral), multivessel disease, presence of left main CAD, and CTO lesions. Detailed procedural parameters were not available as structured data fields in the electronic medical records and could not be systematically retrieved. Pharmacological treatment data were extracted from the hospital's structured electronic prescription and pharmacy dispensing system. Planned dual antiplatelet therapy (DAPT) duration was not consistently documented as a discrete data field and could not be reliably retrieved.

### 2.5 Outcome Definitions

#### 2.5.1 Primary Outcomes

The primary endpoint was major adverse cardiovascular events (MACE), defined as the composite of all‐cause death, non‐fatal MI, stroke, or ischemia‐driven repeat revascularization during follow‐up. MI was diagnosed according to the Fourth Universal Definition (2018) [17]. Stroke was defined as acute focal or global neurological dysfunction caused by vascular injury, confirmed by neuroimaging or neurologist assessment [18].

#### 2.5.2 Secondary Outcomes

Secondary outcomes encompassed cardiovascular, bleeding, and renal events assessed individually during the follow‐up period. Cardiovascular outcomes included target lesion revascularization (TLR) and stent thrombosis. TLR was defined as repeated intervention of the treated segment (5 mm proximal to 5 mm distal to the stent) [19]. Stent thrombosis was classified according to the Academic Research Consortium criteria as definite (angiographic or pathological confirmation) or probable (unexplained death within 30 days after the procedure or target vessel MI without angiographic confirmation) [20, 21]. Bleeding events were evaluated using the Bleeding Academic Research Consortium (BARC) classification, with clinically relevant bleeding defined as BARC type 2–5 [22].

Renal outcomes included CA‐AKI, renal function deterioration, and new‐onset dialysis requirement. CA‐AKI was defined as an absolute increase in serum creatinine ≥ 0.3 mg/dL or a relative increase ≥50% from baseline within 48–72 h after contrast exposure [23]. Renal function deterioration was defined as a sustained decline in eGFR ≥ 30% from baseline during follow‐up, confirmed by at least two consecutive measurements. New‐onset dialysis requirement was defined as initiation of renal replacement therapy (hemodialysis or peritoneal dialysis) during the follow‐up period for patients not previously on dialysis.

### 2.6 Propensity Score Matching

To minimize selection bias, propensity score matching was performed. The propensity score, representing the probability of being classified into the moderate‐to‐severe CKD group, was calculated using multivariable logistic regression incorporating baseline covariates: age, sex, hypertension, diabetes mellitus, prior MI, prior PCI/CABG, atrial fibrillation, heart failure, LVEF, clinical presentation, hemoglobin, multivessel disease, left main disease, and CTO. A 1:1 nearest‐neighbor matching algorithm was employed without replacement, using a caliper width of 0.2 standard deviations of the logit of the propensity score. Covariate balance was assessed using standardized mean differences (SMD), with SMD < 0.1 indicating adequate balance [24]. IPTW with stabilized weights was performed as a sensitivity analysis to assess the robustness of the primary findings. Extreme IPTW weights were truncated at the 1st and 99th percentiles to limit the influence of outlying observations.

### 2.7 Statistical Analysis

Continuous variables are presented as mean ± standard deviation or median (interquartile range) and were compared using Student's *t*‐test or Mann−Whitney *U* test before matching, and paired *t*‐test or Wilcoxon signed‐rank test after matching. Categorical variables are presented as counts (percentages) and were compared using a chi‐square test or a McNemar test. Time‐to‐event analyses were performed using the Kaplan−Meier method with log‐rank test comparison. Cox proportional hazards models with robust sandwich standard errors clustered by matched pairs were used to estimate hazard ratios (HR) with 95% confidence intervals (CI), accounting for matched pairs. The proportional hazards assumption was verified using Schoenfeld residuals. All analyses were performed using SPSS 26.0 and R 4.2.0. Two‐sided *p* < 0.05 was considered statistically significant.

## 3 Results

### 3.1 Study Population and Baseline Characteristics

Between January 2020 and January 2025, a total of 287 consecutive patients underwent PCI with DES implantation at our institution. After applying the exclusion criteria, 87 patients were excluded. The final study cohort comprised 200 patients, of whom 126 (63.00%) were classified into the non‐CKD/mild CKD group (eGFR ≥ 60 mL/min/1.73 m^2^) and 74 (37.00%) into the moderate‐to‐severe CKD group (eGFR < 60 mL/min/1.73 m^2^). The patient selection process is illustrated in Figure 1.

**Figure 2:**
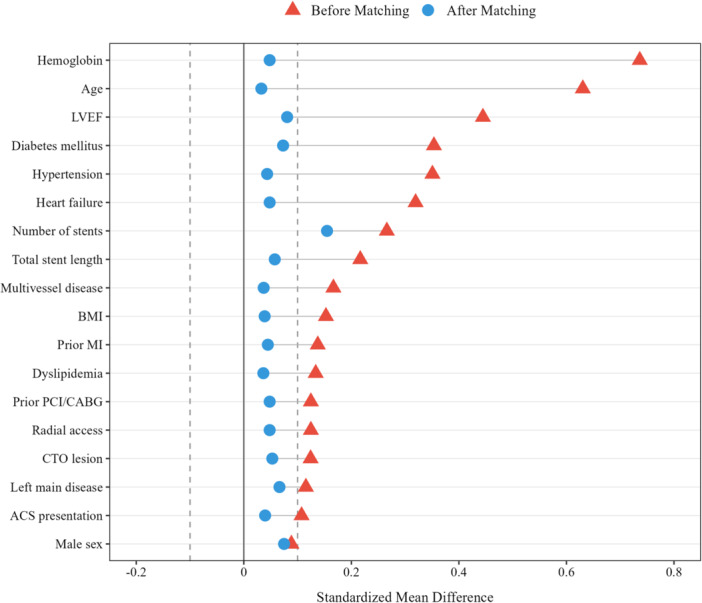
SMDs before and after propensity score matching. The dashed vertical line indicates the threshold of SMD = 0.10, below which covariates are considered well‐balanced. After matching, most covariates achieved SMD < 0.10, with only the number of stents showing a residual SMD slightly above 0.10. BMI, body mass index; CABG, coronary artery bypass grafting; CTO, chronic total occlusion; LVEF, left ventricular ejection fraction; MI, myocardial infarction; PCI, percutaneous coronary intervention; PS, propensity score; SMD, standardized mean difference; SD, standard deviation.

As shown in Table 1, before matching, significant differences were observed between the two groups in several baseline characteristics. Patients with moderate‐to‐severe CKD were older (70.91 ± 9.55 vs. 64.42 ± 11.01 years, *p* < 0.001), had higher prevalence of hypertension (82.43% vs. 67.46%, *p* = 0.033), diabetes mellitus (51.35% vs. 34.13%, *p* = 0.025), and heart failure (22.97% vs. 11.11%, *p* = 0.042), and had lower LVEF (52.14 ± 10.06% vs. 56.42 ± 9.17%, *p* = 0.002) and hemoglobin levels (119.06 ± 18.86 vs. 132.54 ± 17.75 g/L, *p* < 0.001) compared with the non‐CKD/mild CKD group. Procedural and angiographic characteristics were similar between groups, with no significant differences in access site, multivessel disease, left main disease, or CTO lesion prevalence (all *p* > 0.05). After 1:1 propensity score matching, 55 well‐matched pairs were generated. The propensity score model demonstrated good discriminative performance (C‐statistic = 0.79). Before matching, mean propensity scores were 0.28 ± 0.20 in the non‐CKD/mild CKD group and 0.52 ± 0.22 in the moderate‐to‐severe CKD group, with a common support region of 0.17 to 0.83. After matching, propensity scores were well balanced between groups (0.42 ± 0.20 vs. 0.44 ± 0.18). For the IPTW analysis, stabilized weights were truncated at the 1st and 99th percentiles; four patients (2.0%) required truncation with negligible impact on mean weight estimates. After matching, most covariates achieved good balance, with only the number of stents showing a residual SMD above 0.1 (SMD = 0.155), as illustrated in Figure 2.

**Table 1:** Baseline characteristics before and after propensity score matching.

Variable	Before matching				After matching			
	Non‐CKD/mild CKD (*n* = 126)	Moderate‐to‐severe CKD (*n* = 74)	*p*	SMD	Non‐CKD/mild CKD (*n* = 55)	Moderate‐to‐severe CKD (*n* = 55)	*p*	SMD
Age, years	64.42 ± 11.01	70.91 ± 9.55	<0.001	0.630	68.96 ± 9.97	69.27 ± 9.20	0.870	0.033
Male sex, *n* (%)	82 (65.08)	45 (60.81)	0.650	0.088	35 (63.64)	33 (60.00)	0.832	0.075
BMI, kg/m^2^	25.32 ± 3.50	25.87 ± 3.69	0.295	0.153	25.54 ± 3.55	25.68 ± 3.64	0.844	0.039
Hypertension, *n* (%)	85 (67.46)	61 (82.43)	0.033	0.351	42 (76.36)	43 (78.18)	1.000	0.043
Diabetes mellitus, *n* (%)	43 (34.13)	38 (51.35)	0.025	0.354	24 (43.64)	26 (47.27)	0.845	0.073
Dyslipidemia, *n* (%)	58 (46.03)	39 (52.70)	0.444	0.134	27 (49.09)	28 (50.91)	1.000	0.036
Prior MI, *n* (%)	22 (17.46)	17 (22.97)	0.444	0.138	11 (20.00)	12 (21.82)	1.000	0.045
Prior PCI/CABG, *n* (%)	18 (14.29)	14 (18.92)	0.507	0.125	9 (16.36)	10 (18.18)	1.000	0.048
Heart failure, *n* (%)	14 (11.11)	17 (22.97)	0.042	0.319	9 (16.36)	10 (18.18)	1.000	0.048
LVEF, %	56.42 ± 9.17	52.14 ± 10.06	0.002	0.445	54.18 ± 9.22	53.42 ± 9.65	0.675	0.081
Clinical presentation, *n* (%)	84 (66.67)	53 (71.62)	0.568	0.107	38 (69.09)	39 (70.91)	1.000	0.040
Stable coronary artery disease	42 (33.33)	21 (28.38)			17 (30.91)	16 (29.09)		
Acute coronary syndrome	84 (66.67)	53 (71.62)			38 (69.09)	39 (70.91)		
Hemoglobin, g/L	132.54 ± 17.75	119.06 ± 18.86	<0.001	0.736	123.81 ± 16.44	123.00 ± 17.31	0.780	0.048
eGFR, mL/min/1.73 m^2^	82.45 ± 14.64	42.36 ± 12.58	<0.001	2.937	76.28 ± 12.56	44.52 ± 11.97	<0.001	2.588
Multivessel disease, *n* (%)	68 (53.97)	46 (62.16)	0.326	0.167	32 (58.18)	33 (60.00)	1.000	0.037
Left main disease, *n* (%)	8 (6.35)	7 (9.46)	0.597	0.115	4 (7.27)	5 (9.09)	1.000	0.066
CTO lesion, *n* (%)	15 (11.90)	12 (16.22)	0.518	0.124	7 (12.73)	8 (14.55)	1.000	0.053
Number of stents	1.53 ± 0.65	1.72 ± 0.73	0.067	0.266	1.56 ± 0.69	1.67 ± 0.72	0.436	0.155
Total stent length, mm	32.45 ± 19.03	36.77 ± 20.79	0.136	0.217	34.13 ± 19.39	35.27 ± 20.35	0.777	0.058
Radial access, *n* (%)	108 (85.71)	60 (81.08)	0.507	0.125	46 (83.64)	45 (81.82)	1.000	0.048

*Note:* Values are presented as mean ± SD or *n* (%). SMD < 0.1 indicates good balance.

Abbreviations: BMI, body mass index; CABG, coronary artery bypass grafting; CKD, chronic kidney disease; CTO, chronic total occlusion; eGFR, estimated glomerular filtration rate; LVEF, left ventricular ejection fraction; MI, myocardial infarction; PCI, percutaneous coronary intervention; SMD, standardized mean difference.

### 3.2 Primary Efficacy Outcomes

**Figure 3:**
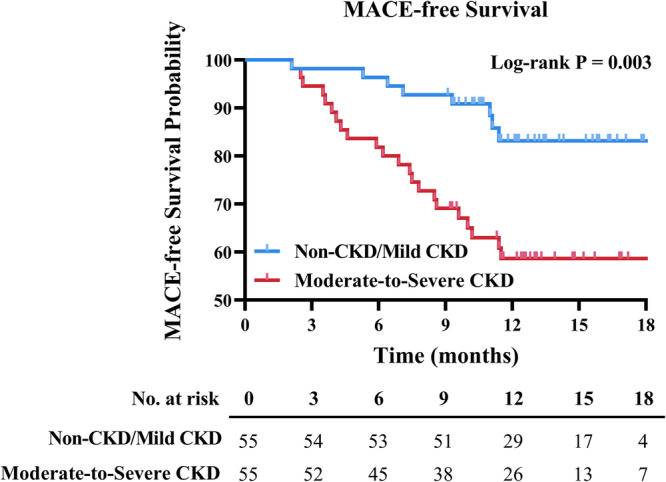
Kaplan−Meier Curves for MACE‐Free Survival. Kaplan−Meier curves depicting MACE‐free survival in patients with moderate‐to‐severe CKD versus non‐CKD/mild CKD following DES implantation. The hazard ratio was calculated using Cox proportional hazards models with robust sandwich standard errors clustered by matched pairs in the propensity score‐matched cohort. CKD, chronic kidney disease; MACE, major adverse cardiovascular events.

The median follow‐up duration was 13.4 months (interquartile range: 11.4–16.8 months). The Kaplan−Meier curves for MACE‐free survival are presented in Figure 3, demonstrating a significantly lower MACE‐free survival in patients with moderate‐to‐severe CKD (log‐rank *p* = 0.003). During the follow‐up, the MACE‐free survival rate was 85.5% in the non‐CKD/mild CKD group versus 60.0% in the moderate‐to‐severe CKD group.

In Cox regression with robust sandwich standard errors clustered by matched pair, moderate‐to‐severe CKD was associated with higher MACE risk compared with non‐CKD/mild CKD (HR 3.20, 95% CI 1.36–7.53; *p* = 0.008). Among individual MACE components, all‐cause death showed an elevated risk in patients with moderate‐to‐severe CKD (*p* > 0.05), which approached statistical significance. The risks of non‐fatal MI, stroke, and ischemia‐driven repeat revascularization were numerically higher in the moderate‐to‐severe CKD group but did not reach statistical significance (all *p* > 0.05) (Table 2).

**Table 2:** Primary outcomes in the propensity score‐matched cohort.

Outcome	Non‐CKD/Mild CKD (*n* = 55)	Moderate‐to‐severe CKD (*n* = 55)	HR	95% CI	*p*
MACE	8 (14.55)	22 (40.00)	3.20	1.36–7.53	0.008
*Components of MACE*					
All‐cause death	2 (3.64)	10 (18.18)	4.44	0.98–20.06	0.052
Non‐fatal MI	2 (3.64)	5 (9.09)	2.28	0.41–12.78	0.350
Stroke	1 (1.82)	3 (5.45)	2.46	0.29–21.20	0.411
Ischemia‐driven repeat revascularization	3 (5.45)	10 (18.18)	2.76	0.70–10.85	0.146

*Note:* Values are presented as *n* (%). Hazard ratios were calculated using Cox proportional hazards models with robust sandwich standard errors clustered by matched pairs, with the non‐CKD/mild CKD group as the reference.

Abbreviations: CI, confidence interval; CKD, chronic kidney disease; HR, hazard ratio; MACE, major adverse cardiovascular events; MI, myocardial infarction.

### 3.3 Secondary Outcomes

Pharmacological treatment is detailed in Supporting Information S1: Table 1. Use of aspirin, P2Y12 inhibitor, statin, β‐blocker, and angiotensin‐converting enzyme inhibitor/angiotensin receptor blocker (ACEI/ARB) was comparable between groups, while SGLT2 inhibitor use was more frequent in the moderate‐to‐severe CKD group in the overall cohort (33.8% vs. 14.3%, *p* = 0.002) but not after matching (*p* = 0.665).

The secondary outcomes results are summarized in Table 3. Among cardiovascular outcomes in the matched cohort, TLR occurred in six patients (10.91%) in the moderate‐to‐severe CKD group and two patients (3.64%) in the non‐CKD/mild CKD group (*p* > 0.05). Definite or probable stent thrombosis was observed in three patients (5.45%) in the moderate‐to‐severe CKD group versus one patient (1.82%) in the non‐CKD/mild CKD group (*p* > 0.05). Neither of these differences reached statistical significance. Regarding bleeding outcomes, BARC type 2–5 bleeding events occurred more frequently in patients with moderate‐to‐severe CKD (12 patients, 21.82%) than in those without CKD (eight patients, 14.55%), yielding an HR of 1.28 (*p* > 0.05). This difference did not reach statistical significance.

**Table 3:** Secondary outcomes in the propensity score‐matched cohort.

Outcome	Non‐CKD/mild CKD (*n* = 55)	Moderate‐to‐Severe CKD (*n* = 55)	HR	95% CI	*p*
*Cardiovascular outcomes*					
Target lesion revascularization	2 (3.64)	6 (10.91)	2.39	0.55–10.35	0.245
Stent thrombosis (definite/probable)	1 (1.82)	3 (5.45)	2.52	0.28–22.47	0.408
Bleeding outcomes					
BARC type 2–5 bleeding	8 (14.55)	12 (21.82)	1.28	0.57–2.84	0.548
*Renal outcomes*					
Contrast‐associated AKI	3 (5.45)	12 (21.82)	3.52	1.13–11.00	0.030
Renal function deterioration (eGFR decline ≥ 30%)	2 (3.64)	11 (20.00)	4.67	1.18–18.46	0.028
New‐onset dialysis	0 (0.00)	5 (9.09)	—	—	0.057

*Note:* Values are presented as *n* (%). Hazard ratios were calculated using Cox proportional hazards models with robust sandwich standard errors clustered by matched pairs, with the non‐CKD/mild CKD group as the reference. For new‐onset dialysis, *p* value was calculated using Fisher's exact test due to zero events in the reference group.

Abbreviations: AKI, acute kidney injury; BARC, Bleeding Academic Research Consortium; CI, confidence interval; CKD, chronic kidney disease; eGFR, estimated glomerular filtration rate; HR, hazard ratio.

For renal outcomes, CA‐AKI occurred in 12 patients (21.82%) in the moderate‐to‐severe CKD group compared with 3 patients (5.45%) in the non‐CKD/mild CKD group and was significantly increased (*p* = 0.030). Renal function deterioration defined as ≥30% decline in eGFR during follow‐up was observed in 11 patients (20.00%) in the moderate‐to‐severe CKD group versus 2 patients (3.64%) in the non‐CKD/mild CKD group, and this difference reached statistical significance (*p* = 0.028). New‐onset dialysis occurred only in the moderate‐to‐severe CKD group (5 patients, 9.09%) and did not reach statistical significance (*p* = 0.057).

### 3.4 Sensitivity Analysis

To assess the robustness of the primary findings, IPTW analysis was performed in the entire pre‐matching cohort of 200 patients. The results are summarized in Table 4. After applying stabilized inverse probability weights, the association between moderate‐to‐severe CKD and MACE remained significant (*p* < 0.001), consistent with the PSM results. The IPTW analysis showed a significant association with CA‐AKI (*p* = 0.001), while all‐cause death did not reach statistical significance (*p* > 0.05).

**Table 4:** Sensitivity analysis: comparison of PSM and IPTW results.

Outcome	PSM analysis (*n* = 110)			IPTW analysis (*n* = 200)		
	HR	95% CI	*p*	Weighted HR	95% CI	*p*
MACE	3.20	1.36–7.53	0.008	4.43	1.88–10.45	<0.001
All‐cause death	4.44	0.98–20.06	0.052	4.71	0.99–22.40	0.051
Non‐fatal MI	2.28	0.41–12.78	0.350	3.90	0.92–16.58	0.066
BARC type 2–5 bleeding	1.28	0.57–2.84	0.548	3.10	1.25–7.73	0.015
Contrast‐associated AKI	3.52	1.13–11.00	0.030	7.06	2.15–23.14	0.001

*Note:* Hazard ratios compare moderate‐to‐severe CKD versus non‐CKD/mild CKD groups. PSM analysis used Cox proportional hazards models with robust sandwich standard errors clustered by matched pairs. IPTW analysis used stabilized weights in the full cohort.

Abbreviations: AKI, acute kidney injury; BARC, Bleeding Academic Research Consortium; CI, confidence interval; HR, hazard ratio; IPTW, inverse probability of treatment weighting; MACE, major adverse cardiovascular events; MI, myocardial infarction; PSM, propensity score matching.

## 4 Discussion

The present study provides an integrated characterization of cardiovascular, bleeding, and renal risk associated with moderate‐to‐severe CKD following contemporary DES implantation, with propensity score matching and IPTW sensitivity analysis employed to strengthen comparability within the observational design. We found that moderate‐to‐severe CKD (eGFR < 60 mL/min/1.73 m^2^) was independently associated with a 3.20‐fold increased risk of MACE compared with preserved kidney function. Additionally, patients with CKD experienced significantly higher rates of renal function deterioration during follow‐up, alongside a significantly higher risk of CA‐AKI and a numerically elevated bleeding risk. These associations were consistent across both PSM and IPTW analyses, although the magnitude of association differed for several outcomes; this pattern supports the robustness of the overall findings within the constraints of the observational design. Collectively, these results may position moderate‐to‐severe CKD as a clinically important marker of residual cardiovascular and cardiorenal risk in patients undergoing DES implantation.

The elevated cardiovascular risk observed in CKD patients following DES implantation in our study aligns with findings from prior investigations. A pooled analysis of randomized trials by Baber et al. demonstrated that women with CKD undergoing DES implantation had substantially higher rates of adverse events, including mortality and stent thrombosis [25]. Similarly, a registry study by Shenoy et al. reported that CKD was associated with higher mortality and MACE following DES placement [7]. Our findings extend this evidence by employing rigorous propensity score matching to account for baseline differences in comorbidities, thereby providing more reliable estimates of the independent association between CKD and post‐PCI outcomes. The magnitude of risk elevation we observed (HR 3.20 for MACE) is consistent with prior reports, suggesting that kidney function may represent a critical predictor of prognosis in this population. The simultaneous elevation of ischemic, hemorrhagic, and renal risk observed in the present study reflects the bidirectional cardiorenal vulnerability of this population, in which accelerated atherosclerosis, endothelial dysfunction, and chronic systemic inflammation converge to amplify both procedural and long‐term risk.

Several pathophysiological mechanisms underlie the heightened cardiovascular risk in CKD patients. Uremic toxins, including indoxyl sulfate and p‐cresyl sulfate, accumulate as kidney function declines and exert direct deleterious effects on the vascular endothelium [26, 27]. These toxins promote endothelial dysfunction, inflammation, and oxidative stress, accelerating atherosclerotic progression and plaque instability. Additionally, tryptophan‐derived uremic toxins have been shown to induce tissue factor expression through the aryl hydrocarbon receptor pathway, potentially contributing to a prothrombotic state [28, 29]. The vascular calcification commonly observed in CKD patients may further complicate coronary interventions through greater procedural complexity and suboptimal stent expansion or apposition [30]. These factors are biologically consistent with the increased susceptibility to ischemic complications, including stent thrombosis and target lesion failure, observed in this population. Paradoxically, CKD patients simultaneously face elevated bleeding risk following PCI. We observed numerically higher rates of BARC type 2–5 bleeding in the moderate‐to‐severe CKD group, though this did not reach statistical significance (HR 1.28, *p* = 0.548) and is presented as an exploratory observation. Platelet dysfunction in uremia, characterized by impaired aggregation and prolonged bleeding time, contributes to this vulnerability [9, 31].

Renal outcomes represent an important but often underappreciated dimension of post‐PCI prognosis. We found that moderate‐to‐severe CKD was associated with a statistically significant increase in renal function deterioration (eGFR decline ≥ 30%; *p* = 0.028). CA‐AKI was observed more frequently in the moderate‐to‐severe CKD group and reached statistical significance in the matched analysis, whereas new‐onset dialysis did not reach statistical significance. These findings are clinically relevant given the established link between CA‐AKI and long‐term adverse outcomes. Mohebi et al. demonstrated that CA‐AKI following PCI was associated with increased mortality and MACE during extended follow‐up [32]. Preventive strategies, including adequate hydration, use of iso‐osmolar contrast agents, and minimization of contrast volume, are particularly important in this high‐risk population [33]. Systematic implementation of standardized periprocedural renal protection protocols encompassing hydration optimization, nephrotoxin avoidance, and procedural contrast volume minimization represents a practical, evidence‐based approach to mitigating CA‐AKI risk in patients with moderate‐to‐severe CKD undergoing PCI. Therefore, the CA‐AKI findings should be interpreted as an observed association in a high‐risk CKD population rather than evidence of a direct procedural causal effect.

These findings carry important clinical implications. Patients with moderate‐to‐severe CKD represent a high‐risk subgroup requiring enhanced monitoring and potentially modified treatment strategies following DES implantation. Risk stratification incorporating kidney function should be routinely performed to identify patients who may benefit from intensified surveillance, optimized medical therapy, and careful attention to contrast exposure and bleeding prevention. The EVOLVE Short DAPT study demonstrated the feasibility of abbreviated antiplatelet therapy in selected high bleeding risk patients, though the optimal duration and intensity of antithrombotic treatment in CKD patients remain areas of active investigation [34]. The simultaneous elevation of ischemic and hemorrhagic risk in patients with CKD presents a particular clinical challenge, underscoring the need for individualized antithrombotic regimens tailored to each patient's specific cardiorenal risk profile. Furthermore, structured cardiorenal follow‐up programs incorporating periodic eGFR monitoring, timely nephrology referral, and optimization of guideline‐directed medical therapy may provide a practical framework for the integrated management of cardiovascular and renal risk in patients with moderate‐to‐severe CKD undergoing DES implantation.

Several limitations of this study should be acknowledged. The retrospective single‐center design inherently limits the generalizability of our findings and precludes definitive causal inference. CKD classification relied on a single baseline eGFR measurement rather than the formally required chronicity criterion of more than 3 months, which could not be systematically confirmed in this retrospective design. The explicit exclusion of baseline AKI and the high burden of chronic comorbidities in the eGFR < 60 mL/min/1.73 m^2^ group substantively support the likelihood of true CKD, though residual misclassification cannot be entirely excluded. Despite rigorous propensity score matching, residual confounding from unmeasured variables cannot be excluded. In addition, although most covariates were well balanced after matching, the number of stents retained a residual SMD slightly above 0.10, which may reflect residual procedural or angiographic complexity and could represent a potential source of residual confounding. The relatively modest sample size, particularly in the matched cohort, may have limited statistical power to detect significant differences in individual MACE components and secondary endpoints. Detailed procedural and renal‐protection data, including contrast volume, contrast type, hydration protocols, nephrotoxin exposure, intravascular imaging, and stent type, were unavailable for systematic extraction, representing potential sources of residual confounding for cardiovascular and renal outcomes. Additionally, the median follow‐up of approximately 13 months may not fully capture DES‐related very late events—including very late stent thrombosis, late in‐stent restenosis, and long‐term progressive renal function decline—which typically require ≥3 years of observation to manifest reliably. Future prospective studies with extended follow‐up are needed to confirm the present findings over a longer temporal horizon. Large‐scale, multicenter prospective studies with extended follow‐up are warranted to validate these findings and further elucidate optimal management strategies for CKD patients undergoing coronary revascularization. Additionally, the planned DAPT duration was not consistently documented as a discrete data field and could not be reliably reported. Furthermore, the binary CKD classification (eGFR ≥ 60 vs. <60 mL/min/1.73 m^2^) does not capture the full heterogeneity of KDIGO stages. Exploratory analysis within our cohort, however, found that MACE rates were relatively homogeneous across Stage G3a (27.3%), G3b (30.0%), and G4 (27.3%), with the predominant risk step‐change occurring at the G2–G3a boundary (8.7% vs. 27.3%), supporting the eGFR 60 threshold as the primary prognostic inflection point in this cohort. Formal stage‐level analyses were precluded by limited numbers in advanced stages (G3b–G5 combined, *n* = 42), and KDIGO‐stratified investigation in adequately powered prospective cohorts is warranted.

## 5 Conclusion

In this propensity score‐matched analysis, moderate‐to‐severe CKD was independently associated with higher risks of MACE and renal function deterioration following DES implantation, with findings supported by IPTW sensitivity analysis. These findings support the incorporation of CKD severity into risk stratification for patients undergoing PCI and highlight the potential value of integrated cardiorenal monitoring and individualized management in this high‐risk population. Prospective multicenter studies with extended follow‐up are needed to confirm these associations and inform optimal treatment strategies.

## Author Contributions

**Hongxu Zhu:** conceptualization, methodology, formal analysis, writing – original draft. **Qi Jin:** investigation, data curation, writing – original draft, writing – review and editing. All authors have read and approved the final manuscript.

## Funding

The authors have nothing to report.

## Ethics Statement

This study was approved by the Institutional Review Board of Beijing Haidian Hospital (approval number: M202620). The requirement for informed consent was waived due to the retrospective nature of this study.

## Conflicts of Interest

The authors declare no conflicts of interest.

## Supporting information

Supporting File "clc70387‐sup‐0001‐Supplementary_Table.docx.

## Data Availability

The data that support the findings of this study are available from the corresponding author upon reasonable request. All data generated or analyzed during this study are included in this published article and its supplementary information files.
