# Supplementary material for: Impact of Chronic Kidney Disease Severity on Clinical Outcomes After Drug‐Eluting Stent Implantation: A Propensity Score–Matched Analysis
Source: Clin Cardiol. 2026 Jun 19;49(6):e70387. doi: 10.1002/clc.70387 (PMC13280797; doi:10.1002/clc.70387)
Supplement: Supplementary file 1 — Supporting File "clc70387‐sup‐0001‐Supplementary_Table.docx. [file CLC-49-e70387-s001.docx]

**Supplementary Table S1 Pharmacological Treatment Before and After Propensity Score Matching**

| **Variable** | **Before Matching** | | | | **After Matching** | | | |
| --- | --- | --- | --- | --- | --- | --- | --- | --- |
|  | **Non-CKD/Mild CKD (n=126)** | **Moderate-to-Severe CKD (n=74)** | **P** | **SMD** | **Non-CKD/Mild CKD (n=55)** | **Moderate-to-Severe CKD (n=55)** | **P** | **SMD** |
| Aspirin | 122 (96.8%) | 71 (95.9%) | 0.711 | 0.047 | 54 (98.2%) | 54 (98.2%) | 1.000 | 0.000 |
| P2Y12 inhibitor | 126 (100.0%) | 74 (100.0%) | 1.000 | 0.000 | 55 (100.0%) | 55 (100.0%) | 1.000 | 0.000 |
| OAC | 10 (7.9%) | 10 (13.5%) | 0.305 | 0.181 | 6 (10.9%) | 4 (7.3%) | 0.742 | 0.127 |
| Statin | 126 (100.0%) | 73 (98.6%) | 0.370 | 0.165 | 55 (100.0%) | 54 (98.2%) | 1.000 | 0.193† |
| β-blocker | 88 (69.8%) | 56 (75.7%) | 0.469 | 0.131 | 40 (72.7%) | 42 (76.4%) | 0.827 | 0.083 |
| ACEI/ARB | 96 (76.2%) | 54 (73.0%) | 0.735 | 0.074 | 45 (81.8%) | 39 (70.9%) | 0.262 | 0.259 |
| SGLT2i | 18 (14.3%) | 25 (33.8%) | 0.002 | 0.469 | 13 (23.6%) | 16 (29.1%) | 0.665 | 0.124 |

Data are presented as n (%). P-values for the unmatched cohort were calculated using chi-square test or Fisher’s exact test as appropriate; P-values for the matched cohort were calculated using McNemar’s test. P2Y12 inhibitor data are presented at the overall class level rather than by individual agent. Planned DAPT duration was not consistently documented as a discrete field in the electronic records and could not be reliably reported. ^†^ SMD for statin use should be interpreted with caution given the near-ceiling proportion in both groups (100% vs. 98.2%); the absolute difference corresponds to a single patient.

Abbreviations: OAC, oral anticoagulant; ACEI, angiotensin-converting enzyme inhibitor; ARB, angiotensin receptor blocker; SGLT2i, sodium-glucose cotransporter-2 inhibitor; SMD, standardized mean difference; PSM, propensity score matching.
